# A novel deep learning approach for diagnosing Alzheimer's disease based on eye-tracking data

**DOI:** 10.3389/fnhum.2022.972773

**Published:** 2022-09-09

**Authors:** Jinglin Sun, Yu Liu, Hao Wu, Peiguang Jing, Yong Ji

**Affiliations:** ^1^School of Microelectronics, Tianjin University, Tianjin, China; ^2^Tianjin Key Laboratory of Cerebrovascular and Neurodegenerative Diseases, Department of Neurology, Tianjin Dementia Institute, Tianjin Huanhu Hospital, Tianjin, China; ^3^School of Electrical and Information Engineering, Tianjin University, Tianjin, China

**Keywords:** Alzheimer's disease, deep learning, eye-tracking, artificial intelligence, biomedical application

## Abstract

Eye-tracking technology has become a powerful tool for biomedical-related applications due to its simplicity of operation and low requirements on patient language skills. This study aims to use the machine-learning models and deep-learning networks to identify key features of eye movements in Alzheimer's Disease (AD) under specific visual tasks, thereby facilitating computer-aided diagnosis of AD. Firstly, a three-dimensional (3D) visuospatial memory task is designed to provide participants with visual stimuli while their eye-movement data are recorded and used to build an eye-tracking dataset. Then, we propose a novel deep-learning-based model for identifying patients with Alzheimer's Disease (PwAD) and healthy controls (HCs) based on the collected eye-movement data. The proposed model utilizes a nested autoencoder network to extract the eye-movement features from the generated fixation heatmaps and a weight adaptive network layer for the feature fusion, which can preserve as much useful information as possible for the final binary classification. To fully verify the performance of the proposed model, we also design two types of models based on traditional machine-learning and typical deep-learning for comparison. Furthermore, we have also done ablation experiments to verify the effectiveness of each module of the proposed network. Finally, these models are evaluated by four-fold cross-validation on the built eye-tracking dataset. The proposed model shows 85% average accuracy in AD recognition, outperforming machine-learning methods and other typical deep-learning networks.

## 1. Introduction

Alzheimer's disease is a nonreversible neurodegenerative disease, which involves parts of the brain that control thought, memory, and language and can seriously affect a person's ability to carry out daily activities. It is the most common form of dementia, and subjects with mild cognitive impairment (MCI) have a higher risk of transitioning to dementia (Hishikawa et al., 2016). Presently, the evaluation criteria of cognitive function state are usually quantified through neuropsychological tests, such as the Mini Mental State Examination (MMSE) (Folstein, 1975) and Montreal Cognitive Assessment (MoCA) (Nasreddine et al., 2005). Although these methods have become mainstream diagnostic methods for detecting cognitive impairment in AD, there are still some unavoidable limitations that hinder their wider application. For example, such tests generally need to take 10–15 min to complete in an absolutely quiet space under the guidance of professionally trained medical staff. This will cause a significant consumption both of time and manpower. Moreover, this type of test will easily cause the subjects to be nervous and uneasy, resulting in less objective evaluation scores. Although Alzheimer's disease is considered nonreversible, diagnosis and treatment at an early stage can help to overcome the symptoms and allow more aggressive therapy to prevent progression to dementia (Readman et al., 2021).

As a simple, accurate and non-invasive screening tool, eye-tracking has shown great potential for AD diagnosis in previous research (Chaabouni et al., 2017; Nam et al., 2020; Pavisic et al., 2021; Readman et al., 2021). Since patients with Alzheimer's Disease (PwAD) are usually accompanied by a broad spectrum of oculomotor alterations, a growing body of research provides evidence for some specific eye movements as biomarkers in early AD stages. For example, numerous studies have shown that abnormal saccades are associated with AD (Crawford et al., 2017; Orlosky et al., 2017; Kahana Levy et al., 2018). Experimental designs in such research are generally divided into two categories: prosaccades, which require the user's eyeballs to move toward a presented stimulus, and antisaccades, which require the user's eyeballs to move away from the stimulus. It is noted that AD patients had longer delays in initiating prosaccades and antisaccades compared with controls. Besides, PwAD are more prone to errors in performing the antisaccade task than the presaccade (Kahana Levy et al., 2018). In addition, since cognitive impairment is often accompanied by impaired memory recognition, episodic memory deficits have been well characterized in detecting earlier AD. Converging finding also suggests that eye movement behavior reveals different mnemonic processes, including before or even in the absence of conscious recollection (Hannula et al., 2010; Bueno et al., 2019; Tadokoro et al., 2021).

To explore the episodic memory process in AD, several visuospatial memory eye-tracking tasks, such as the visual short-term memory (VSTM) test (Pertzov et al., 2012; Haque et al., 2020; Pavisic et al., 2021) and visual paired comparison (VPC) test (Haque et al., 2019; Nie et al., 2020), have been designed in current research. For the VSTM task, Pavisic et al. (2021) designed an "object-location" VSTM task with 52 participants. In this task, individuals first viewed a sample array (memory array) of 1 or 3 fractal objects. Then in the test array, they were required to touch the fractal they remembered from the memory array and its original position on the touch-screen. Results focused on two memory measures of task performance: recognition accuracy and localization. By analyzing the experimental results, the researchers found that presymptomatic carriers were less accurate at locating targets than controls for shorter stimulus fixation times. As for the VPC task, it is a recognition memory test that has a proven sensitivity to memory decline. Typically, participants are first presented with a series of visual stimuli for a period of time with no explicit instructions. After a delay, the stimuli are presented again side-by-side with a novel image. Compared to the original image, an object will be added or removed in the new image. Several studies have employed VPC comparison tasks with artificial stimuli (Lagun et al., 2011; Chau et al., 2015) and naturalistic visual stimuli (Crutcher et al., 2009). For example, Chau et al. (2015) conducted the VPC task by presenting PwAD and HCs incorporating artificial stimulus to quantify novelty preference in AD patients by measuring visual scanning behavior. In contrast, Crutcher et al. (2009) assessed the performance of the VPC task to detect MCI under naturalistic scenes. As indicated in review literature (Readman et al., 2021), such tasks combined with eye-tracking technology shows particularly promising results for the distinction between MCI, PwAD, and HCs populations. However, It is worth mentioning that almost the current VPC tasks only concentrate on 2D display, and few studies have used a 3D display form.

Machine-learning-based and deep-learning-based models have been shown to play a crucial role in identifying cognitive function impairment with high sensitivity (Fabrizio et al., 2021; Miltiadous et al., 2021; Murdaca et al., 2021; Rizzo et al., 2022). These models are commonly used to deal with medical imaging such as positron emission computed tomography (PET) or magnetic resonance imaging (MRI), mainly because the feature representations produced by these models can be helpful even if the data is partially missing (Biondi et al., 2017). In addition to medical images, deep-learning models combined with eye tracking technology have also shown good performance in identifying neurological diseases (Biondi et al., 2017; Chaabouni et al., 2017; Jiang and Zhao, 2017). Jiang and Zhao (2017) proposed a Deep Neural Networks (DNN) based model to identify people with Autistic Spectrum Disorder (ASD), using the eye-tracking data in free image viewing. With a fine-tuned VGG-16 network extracting features and a linear Support Vector Machine (SVM) to do the classification, their model achieved an accuracy of 92%. Chaabouni et al. (2017) built a deep-learning architecture to predict the visual attention model of patients with Dementia, and this model achieved a predictive accuracy of 99.27%. However, deep-learning models for AD recognition or classification based on eye movement data are rare in existing studies due to the lack of large-scale eye-tracking datasets. Biondi et al. (2017) developed a deep-learning approach to differentiate between the eye-movements reading behavior of PwAD over HCs. Using a trained autoencoder and a softmax classifier, this model conducted 89.78% of accuracy for identifying PwAD.

The above encouraging results indicate that deep-learning models are promising for understanding the dynamics of eye-movement behavior and its relationship to underlying cognitive impairment processes. However, several limitations have hindered applying such research in broader fields. For example, due to the insufficiency of existing eye-tracking system functions and algorithms in 3D displays, almost all experimental stimuli in the above studies are displayed in two dimensions. Even though it has been pointed out that under the same content, 3D stimuli can stimulate more abundant eye movements and brain activity behaviors than 2D stimuli (González-Zúñiga et al., 2013). Moreover, further research is limited by the lack of the large-scale eye-tracking dataset since the performance of deep-learning-based approaches depends heavily on the training dataset's size.

Motivated by the above limitations, we propose the NeAE-Eye: a nested autoencoder model used to identify the PwAD and HCs with an eye-movement dataset on a designed 3D VPC task. The hypothesis is that using deep learning to identify the key characteristics of the patient's eye behavior during the VPC task may lead to a correct classification, which can be used to infer a diagnosis of AD. Our main contributions to this paper can be summarized as follows:

- We propose a novel deep-learning-based model with nested autoencoder networks to classify the PwAD and the HCs based on their generated gaze heatmaps;
- We constructed a large-scale eye-movement dataset using the designed 3D VPC task, which can be used to analyze the differences in eye movement characteristics of PwAD and HCs under stereoscopic stimuli. To the best of our knowledge, we are the first to design such task stimuli in a stereo format;
- To fully validate model performance, two types of models are designed based on machine learning and deep learning, respectively. In addition, we also conduct ablation experiments to verify the effectiveness of each module of the NeAE-Eye model.

## 2. Materials and methods

In this section, we present the materials and methods used in the study, including participants, study design, data acquisition and preprocessing, the framework of NeAE-Eye and models for comparison, evaluation metrics.

### 2.1. Participants

A total of 108 patients with AD and 102 healthy controls were recruited from September 2020 to September 2021 at the cognitive impairment clinics, Tianjin HuanHu Hospital, Tianjin, China. All participants were 40−92 years of age. Diagnoses for AD were carried out on grounds of the patients' clinical history, neuropsychological examination, and structural imaging. Probable AD was diagnosed according to the criteria of the National Institute on Aging and the Alzheimer Association workgroup (McKhann et al., 2011). Exclusion criteria were as follows: diagnosis of any neurological disease except AD; patients with uncorrected dysfunctions of vision, hearing loss, aphasia, or an inability to complete a clinical examination or scale assessment; history of mental disorders and illicit drug abuse; patients with acute or chronic liver and kidney dysfunction, malignant tumors, or other serious underlying diseases. HCs were recruited from friends and relatives of the patients and had no history of psychiatric or neurological illness or evidence of cognitive decline. Besides, in our research, the PwAD group did not include patients with MCI. In our study, all procedures followed conformed to the World Medical Association Declaration of Helsinki.

### 2.2. Study design

We performed a statistical analysis of eye-tracking data under the VPC task to compare characteristic differences between the PwAD and HCs groups and then determined whether these differences could serve as a screening tool to identify AD.

#### 2.2.1. Calibration procedure

In order to track the participants' gaze more accurately, each participant in the experiment performed a personal calibration with a self-designed 3D eye-tracking system (Sun et al., 2021). The device integrates a stereoscopic playback system and a binocular eye-tracking system to provide users with a comfortable viewing experience while achieving high-sensitivity 3D gaze estimation with an accuracy of 0.66°.

**Figure 1:**
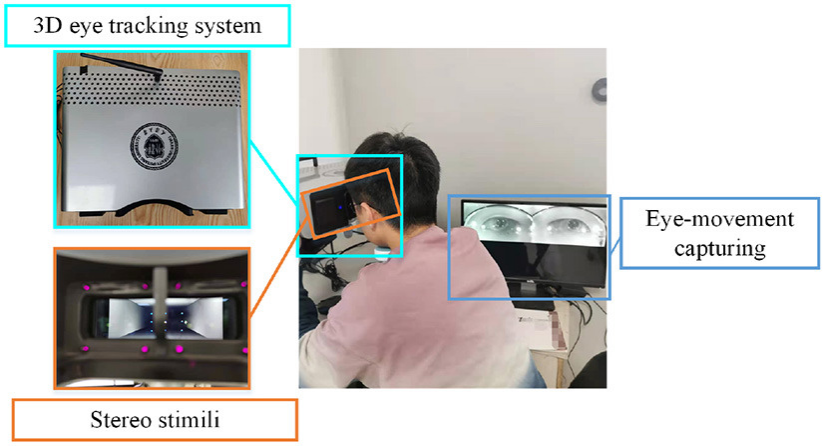
The user-friendly data collection scene with the self-designed 3D eye-tracking system.

During the calibration procedure, each participant is presented with nine points at random locations on the screen, and each mark will be displayed for 3 s. Eye-movement images will be captured for each mark, and gaze points will be tracked based on these images. The data collection scene is shown in Figure 1. The duration of the calibration process is 27 s, and whole experimental session takes a total of 4 min and 27 s.

#### 2.2.2. 3D visual paired comparisons task

**Figure 2:**
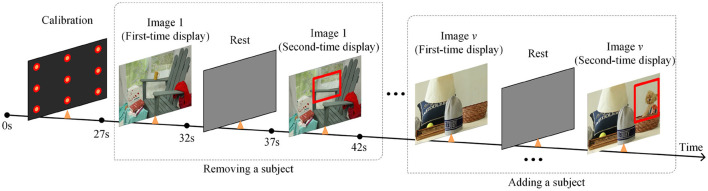
The whole process description of the designed 3D VPC task.

This paper designs a 3D VPC task with stereo stimuli and the whole process is shown as Figure 2. After the calibration process, the task begins with the presentation of a series of images of stereo scenes for a duration of 5 s each. There are a total of 12 images for testing. And then, a similar set of images will be displayed, each with an object added or removed from the original image. Besides, each image is designed or selected to contain 4–8 objects in total, and these stereo images include different scenes, including natural scenes, cartoon scenes, geometric scenes, etc.

During the task, participants will not be given any instructions but only required to freely view the images. Additionally, compared with the images displayed for the first time, the regions where objects are added or deleted in the second time display are defined as the region of interest (RoI). For memory and novelty preference exploration in PwAD, the relative fixation time on the previously displayed images and the novel images will be measured by the eye-tracking system.

### 2.3. Data collection and preprocessing

The resolution of eye movement data collected by the self-designed 3D eye-tracking system is 1,920 x 1,080 pixels, and the sampling rate of the eye camera is 120 Hz. During the task, the subject does not need to wear any equipment but only needs to put his head on the forehead rest. This friendly measurement allows the subjects to relax so that the collected data reflects their normal state.

**Figure 3:**
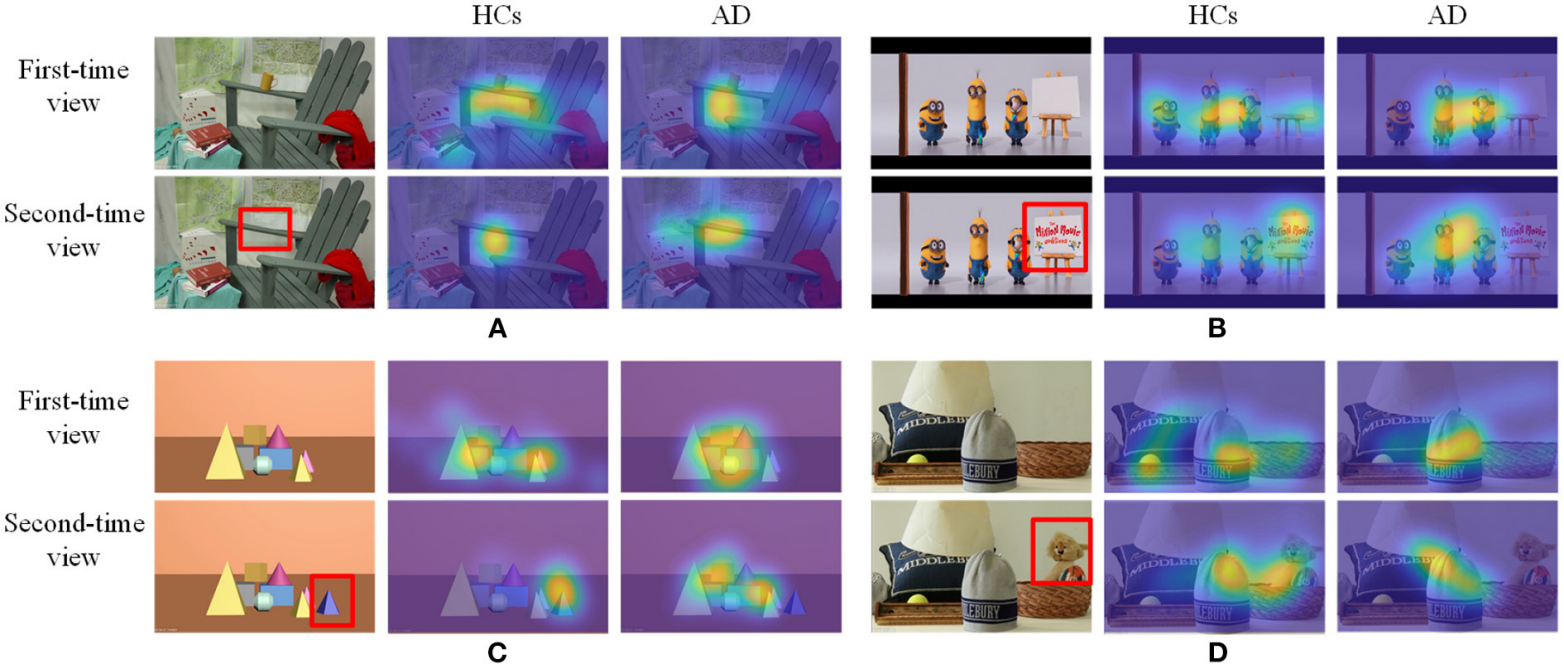
Some samples of the testing images and corresponding fixation heatmaps generated for each group and each time viewing. **(A)** Indoor far-view scene; **(B)** Cartoon scene; **(C)** Geometric scene; **(D)** Indoor near-view scene.

For each image in each group, a fixation map will be constructed by overlaying all estimated fixation points for each subject into a map. Then, the generated fixation map can be smoothed and normalized with a Gaussian kernel to generate the fixation heatmap, as shown in Figure 3. Finally, all fixation heatmaps will be used as the source input for the NeAE-Eye network to extract discriminative features for PwAD and HCs classification.

Since these test images require different degrees of memory function, the degree of difference in the fixation heatmaps between PwAD and HCs is also different. Figure 3 shows four samples with apparent visual differences in the fixation heatmaps between PwAD and HCs. The two-row heatmaps for each image are generated from the first-time view and second-time view fixation data, respectively. As can be observed from the fixation heatmaps in Figure 3, the participants in the HCs group pay attention to both global images and the RoI areas. However, PwAD prefer to look at the central area of the images and pay less attention to the RoI areas than the HCs group. This particular gaze-following phenomenon is further evidence of AD's lack of novelty preference. Therefore, it is sufficient to hypothesize that eye-movement data in the 3D VPC task can reflect the memory activity occurring in the subject's brain. This is of great value in supporting clinical practice in diagnosing AD.

### 2.4. Framework of the NeAE-Eye network

**Figure 4:**
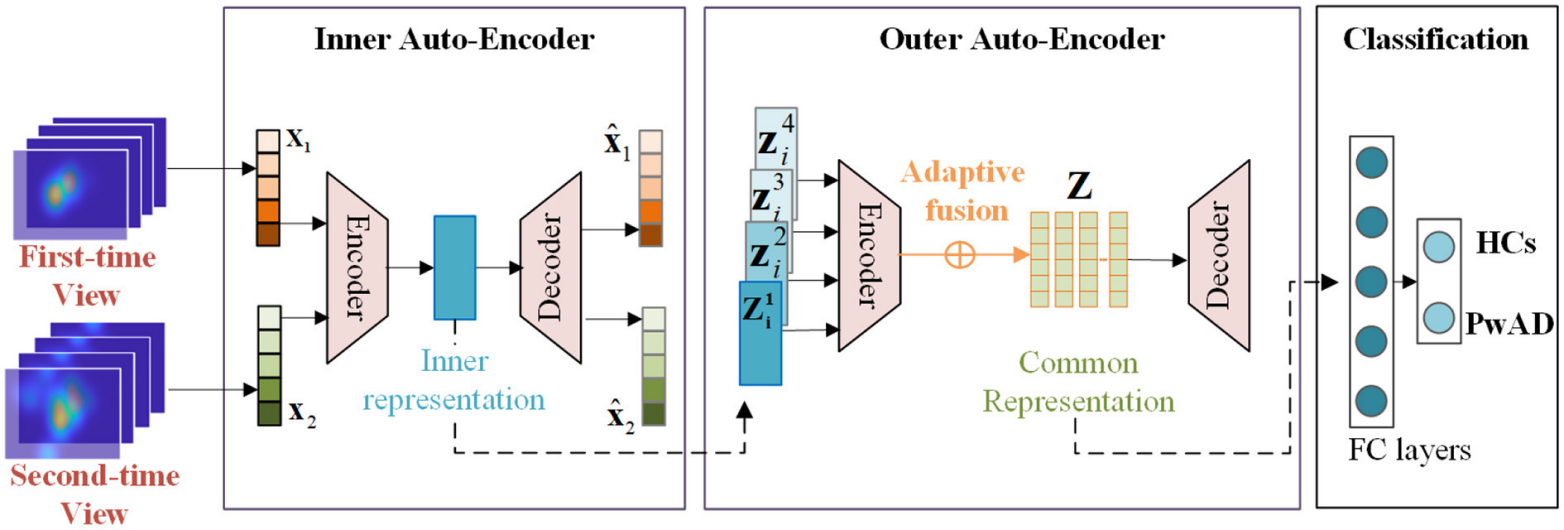
This figure shows the whole workflow of the proposed NeAE-Eye network. The NeAE-Eye model has three main modules, including inner autoencoder module, outer autoencoder module, and classifier module.

In this section, we give a detailed description of the proposed NeAE-Eye model for AD identification, as shown in Figure 4. The model of NeAE-Eye consists of three main modules: inner autoencoder, outer autoencoder, and classifier.

#### 2.4.1. Encoding for the internal representation learning

The key goal of the inner autoencoder module is to learn the image-dependent representation based on the fixation density maps for each test image, which consists of a shallow convolutional network with 8-layers. The first four layers of the shallow convolutional network act as encoders, which is used to extract low-dimensional features of fixation heatmaps. For the last four layers, they act as decoders for reconstructing the original input from internal representations.

For simplicity, given the subject's fixation heatmaps **x**_1_, **x**_2_ for each test image generated when viewing for the first and second time, the processes of the inner encoder **E**_*ae*_*in*_, inner decoder **E**_*de*_*in*_ can be denoted as:


(1) $$\begin{array}{l} \{\begin{array}{l} \text{z}={\text{E}}_{ae\_in}({\text{x}}_{1},{\text{x}}_{2};\beta_{ai})\\ {\hat{\text{x}}}_{1},{\hat{\text{x}}}_{2}={\text{E}}_{de\_in}(\text{z};\beta_{di}) \end{array} \end{array}$$

where **z** is the learned inner representation for a single image and ${\hat{\text{x}}}_{1},{\hat{\text{x}}}_{2}$ are the reconstructed input fixation heatmaps by the decoders. Besides, ***β***_*ai*_, ***β***_*di*_ represent the parameter set for the encoder and decoder layers, respectively. Since the inner representation **z** is supposed to preserve more intrinsic information from the two inputs **x**_1_, **x**_2_, we train this encoding network by minimizing the following reconstruction loss:


(2) $$\begin{array}{l} L_{com1}=\underset{\left\{\beta_{ai},\beta_{di}\right\}}{\text{min}}\frac{1}{2}\overset{2}{\underset{v=1}{\sum }}\|x_{v}-{\hat{\text{x}}}_{v}{\|}_{F}^{2}. \end{array}$$

The reason that we introduce the autoencoder network to extract the inner representation is that there is no supervised information to guide the learning process. Besides, the autoencoder network can extract the intrinsic information from multi-view inputs while filtering out the original high-dimensional features.

#### 2.4.2. Outer encoder for the common representation learning

After obtaining the image-dependent representation, we focus on encoding them into an intact common representation which can be used to highlight the difference of the visual attention mechanism between the PwAD and HCs. To this end, a fully connected neural network (FCNN) is employed in the outer autoencoder module, which consits of the encoder **E**_*ae*_*out*_ and, decoder **E**_*de*_*out*_ and a weight adaptive fusion layer *f*_*map*_.

Specifically, let $\left\{{\text{z}}_{i}^{v}|v\in \left\{1,2,...,V\right\},i\in \left\{1,2,...,N\right\}\right\}$ denotes an image-dependent representation set for the *i*-th subject, where *V* is the total number of the test images and *N* is the number of the total subjects, we treat the *V* test images of the same sample as *V* different views for the degradation network's input. Then, the processes of the outer autoencoder can be denoted as:


(3) $$\begin{array}{l} \{\begin{array}{c} \;\{{\tilde{\text{z}}}_{i}^{v}\}={\text{E}}_{ae\_out}(\{{\text{z}}_{i}^{v}\};\beta_{ao})\\ \left\{{\hat{\text{z}}}_{i}^{v}\}={\text{E}}_{de\_out}(Z_{i};\beta_{do}\right) \end{array} \end{array}$$

where $\left\{{\tilde{\text{z}}}_{i}^{v}\right\}$ is a low-dimensional representation of $\left\{{\text{z}}_{i}^{v}\right\}$. Besides, the β_*ao*_, β_*do*_ are the parameter sets of the outer encoder and decoder.

After getting the latent representation $\left\{{\tilde{\text{z}}}_{i}^{v}\right\}$ of *V* views from the encoder network, a weight adaptive fusion layer is introduced to seek the final intact common representation **Z**_*i*_ of multi-views:


(4) $$\begin{array}{l} {\text{Z}}_{i}=f_{map}\left({\tilde{\text{z}}}_{i}^{1},{\tilde{\text{z}}}_{i}^{2},...,{\tilde{\text{z}}}_{i}^{V};\beta_{m}\right),i\in \left(1,N\right), \end{array}$$

where *f*_*map*_(·) denotes the fusion function and the β_*m*_ = {β_1_, ..., β_*V*_} is a group learnable parameter of the fusion layer. Likewise, we believe that the common representations learned by different views of the same sample should be as identical as possible to reflect consistency between views. Therefore, the objective of the outer autoencoder can be defined as


(5) $$\begin{array}{l} L_{com2}=\underset{\left\{\beta_{ao},\beta_{do},\beta_{m}\right\}}{\min}\frac{1}{2}\left(\overset{V}{\underset{v=1}{\sum }}\|{\text{z}}^{v}-{\tilde{\text{z}}}^{v}{\|}_{F}^{2}+\|\text{Z}-\overset{V}{\underset{v=1}{\sum }}\beta_{m}{\tilde{\text{z}}}^{v}{\|}_{F}^{2}\right). \end{array}$$

#### 2.4.3. Classification on the common representation

Based on the learned intact common representation {**Z**_*i*_|*i* ∈ (1, *N*)} of *N* subjects, the final binary classification will be processed by a 3-layers FC network with a Cross-Entropy loss function:


(6) $$\begin{array}{l} L_{cls}=\overset{N}{\underset{i=\text{1}}{\sum }}y_{i}\log\left(\sigma \left({\text{Z}}_{i}\right)\right)+\left(\text{1}-y_{i}\right)\log\left(\text{1}-\sigma \left({\text{Z}}_{i}\right)\right), \end{array}$$

where *y*_*i*_ is the label of the *i* th subject.

In conclusion, the total loss function of the whole NeAE-Eye model can be represented as *L* = *L*_*cls*_ + *L*_*com*1_ + *L*_*com*2_.

### 2.5. Models for comparison

**Figure 5:**
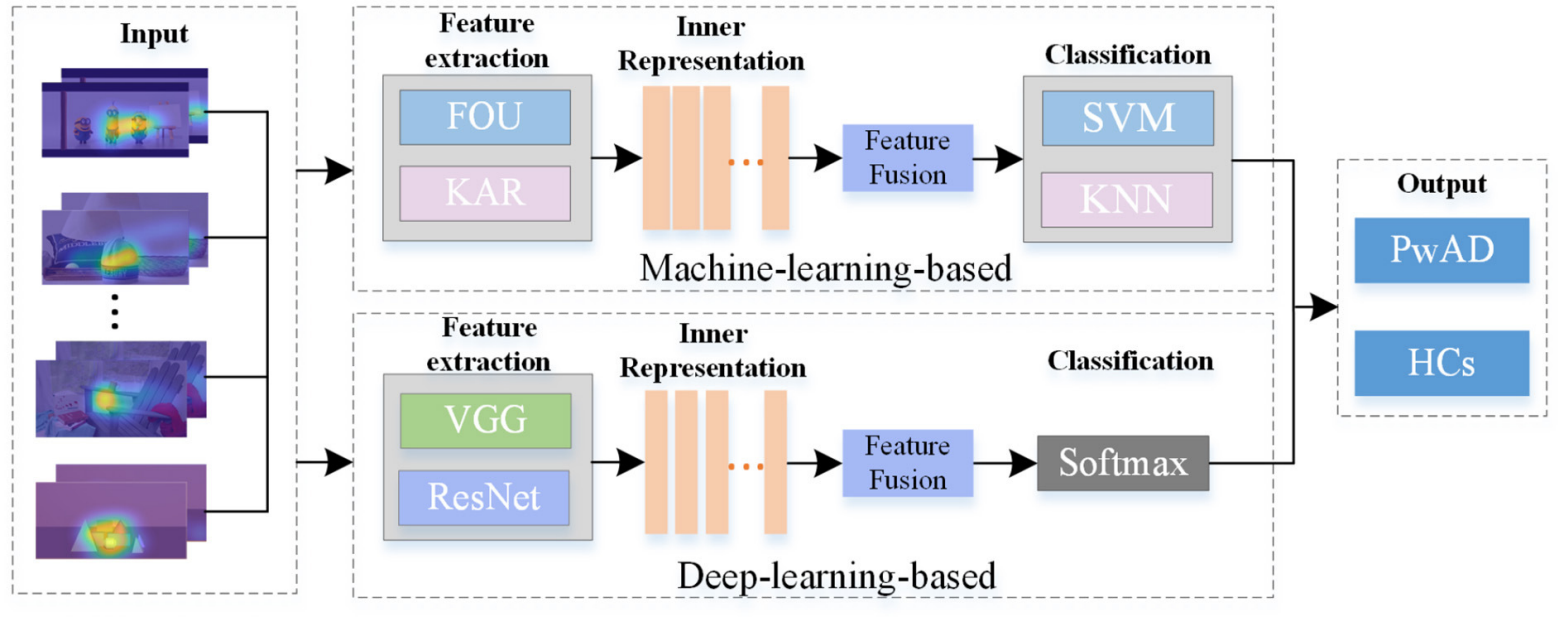
The main framework of the models for comparison. For the machine-learning-based methods, we employ the FOU and KAR for feature extraction and the SVM and KNN for binary classification, respectively; And for deep-learning-based methods, we use VGG-16 network and Resnet-18 network to extract the features and use FC networks for classification.

The major framework of some typical models for comparison has been shown in Figure 5, which consists of three main modules, including feature extraction, feature fusion and classification. These comparative approaches can be divided into machine-learning-based models and deep-learning-based models. For approaches with machine-learning, we first employ two different feature descriptors: the Fourier coefficients for two-dimensional shape descriptors (FOU) and Karhunen-Loeve coefficients (KAR), for image-dependent feature extraction. And then, in the stage of classification, both the SVM and K-Nearest Neighbors (KNN) are used to classify the two groups. As for approaches with deep-learning, we adopt the widely used VGG-16 network and Resnet-18 network to achieve the features extraction. And then, we still use the same FC network as the NeAE-Eye model for the final classification. For both types of approaches, we choose to connect all feature vectors to obtain the common representation. Moreover, we set the proposed NeAE-Eye as the base model and set up the comparative models as different combinations of different feature extraction modules and classification modules, such as FOU_SVM, KAR_KNN, VGG16_FC, etc.

### 2.6. Evaluation metrics

In this paper, we introduce four assessment criteria: accuracy, precision, recall and F1-score, to evaluate the performance of the model, which are calculated according to the Equations 7–10 specified below.


(7) $$\begin{array}{l} A\text{ccuracy =}\frac{TP+TN}{TP+TN+FP+FN} \end{array}$$

(8) $$\begin{array}{l} P\text{recision =}\frac{TP}{TP+FP} \end{array}$$

(9) $$\begin{array}{l} \text{Recall =}\frac{TN}{TP+FN} \end{array}$$

(10) $$\begin{array}{l} \text{F1=}2\times \frac{Precision\times Recall}{Precision+Recall} \end{array}$$

where *TP, FP, FN, TN* are the calculated true positives, false positives, false negatives, and true negatives separately. In this paper, we take PwAD as the positive samples and HCs as the negative samples. Based on the assessment metric, we also derived the receiver operating characteristic (ROC) curves and area under the curve (AUC) values to show specific details for the experimental results.

## 3. Results

### 3.1. Classification with statistical-based method

To initially validate the feature distribution of the constructed eye-tracking dataset, we first roughly classified PwAD and HCs using a traditional statistics-based method. In this method, we first introduce the classical principal component analysis (PCA) for feature selection from the fixation heatmaps, then use the Naive Bayes classifier to complete the classification process. Since the initial dimension of the generated heatmap is 768 (32 × 24), we set the feature dimensions selected by PCA as *m* = 20, *m* = 40, *m* = 60, *m* = 80 and *m* = 100 for comparative analysis.

**Figure 6:**
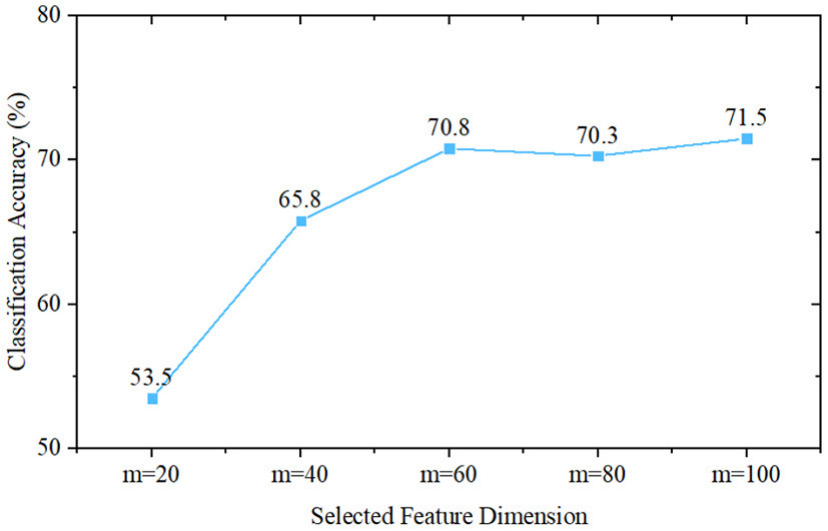
Classification performance of Naive Bayes classifier-based methods, where *m* is the selected feature dimension of the principal component analysis.

The classification performance of these five different settings is shown in Figure 6. It can be seen from the experimental results that when *m* = 20, the classification accuracy is 53.5%. As *m* increases, the classification accuracy first increases, and when *m*≥60, it remains almost unchanged. The best performance is obtained when *m* = 100, and the classification accuracy is 71.5%. This result also implies that there are indeed differences between AD and HCs eye movement features, which can provide a mathematical basis for machine-learning-based and deep-learning-based models.

### 3.2. Classification results of different models

We evaluate the performance of each model by applying four-fold cross-validation to assess model robustness. The classification results of different models are displayed in Table 1. From the Table 1, the NeAE_Eye model outperforms all other classifier models, showing 85% mean accuracy, followed by Resnet18_FC and VGG16_FC with the accuracy of 81 and 80%, respectively. Besides, by comparing the models with traditional feature descriptors, we can find that the model combined with the SVM classifier outperforms that combined with KNN. For example, the FOU_SVM model achieves 0.71 ± 0.03 mean precision, 0.78 ± 0.04 mean recall, 0.74 ± 0.03 mean F1-score, and 0.74 ± 0.04 mean accuracy, which is higher than that of FOU_KNN. Furthermore, the average performances of the deep-learning-based models are better than that of machine-learning-based methods.

**Table 1:** Results comparison with the state-of-the-art models.

**Categories**	**Models**	**Mean precision**	**Mean recall**	**Mean F1-score**	**Mean accuracy**
Machine-learning				FOU_SVM	0.71 ± 0.03	0.78 ± 0.04	0.74 ± 0.03	0.74 ± 0.04
FOU_KNN	0.63 ± 0.02	0.65 ± 0.03	0.64 ± 0.03	0.63 ± 0.07
KAR_SVM	0.76 ± 0.04	0.80 ± 0.02	0.78 ± 0.04	0.72 ± 0.03
KAR_KNN	0.65 ± 0.04	0.68 ± 0.05	0.66 ± 0.05	0.69 ± 0.04
Deep-learning			VGG16_FC	0.82 ± 0.05	0.92 ± 0.02	0.87 ± 0.03	0.80 ± 0.02
Resnet18_FC	0.85 ± 0.03	0.87 ± 0.01	0.86 ± 0.04	0.81 ± 0.03
NeAE-Eye	0.87 ± 0.04	0.89 ± 0.04	0.88 ± 0.04	0.85 ± 0.05

**Figure 7:**
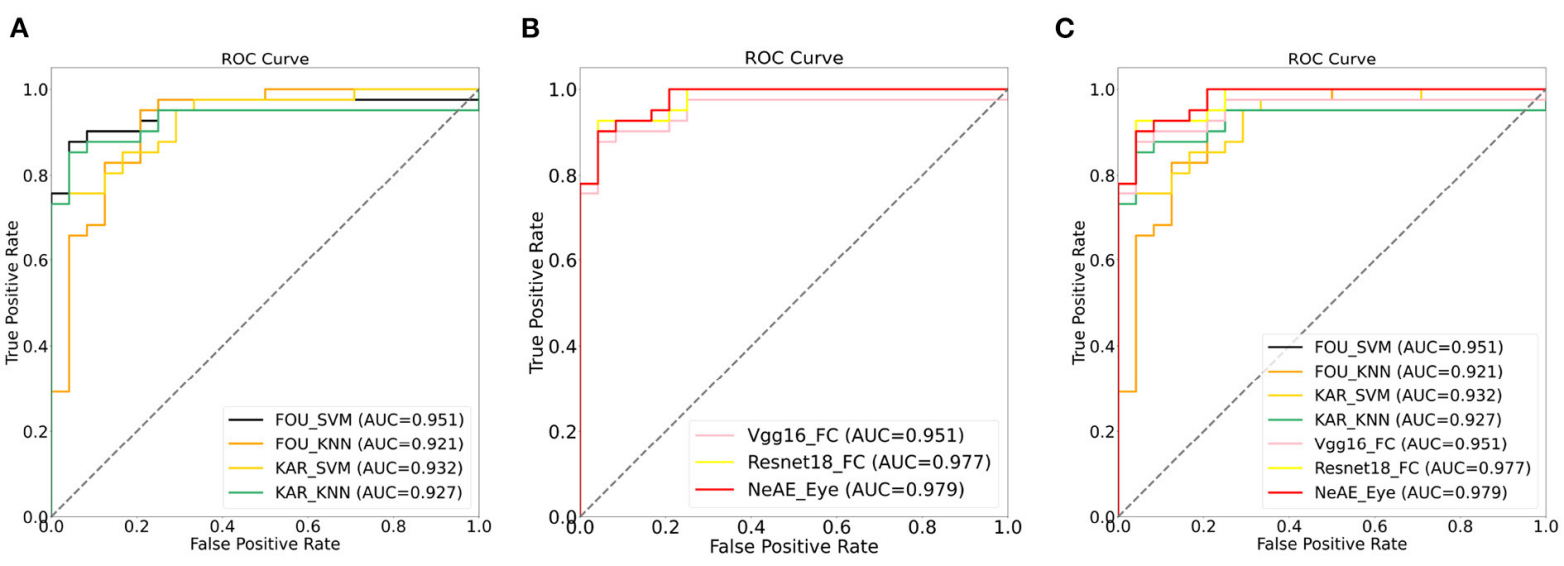
Receiver operation characteristic (ROC) curves: **(A)** ROC curves of models with machine-learning models; **(B)** ROC curves of models with deep-learning networks; **(C)** ROC curves of all models.

Figure 7 shows a comparison between the ROC curves of the approaches with machine-learning models (Figure 7A) and approaches with deep-learning networks (Figure 7B). For these two category models, the achieved AUCs range from 0.921 to 0.951 and 0.951 to 0.977, respectively. The methods with machine-learning models show mean AUCs of 0.933. For the models with deep-learning networks, the mean AUCs is 0.969, which scores 0.036 higher than that of machine-learning models.

### 3.3. Ablation experiment for the modules in NeAE-Eye model

To more comprehensively evaluate the performance of the proposed model, we design an ablation experiment to verify the importance of each module of the model. Since the NeAE-Eye model consists of the inner encoder (*EN*_*in*_), the inner decoder (*DE*_*in*_), the outer encoder (*EN*_*out*_), the outer decoder (*DE*_*out*_) and the weight adaptive fusion layer *f*_*map*_, we conduct the ablation studies of different module combinations to assess their efficacy.

As shown in Table 2, we set the combination of the inner encoder and the outer encoder (*EN*_*in*__*EN*_*out*_) as the basic reference. Then, by successively adding the inner decoder, the out decoder and the weight adaptive fusion layer *f*_*map*_, we compare their classification effectiveness also using the mean precision, recall, F1-score and accuracy. From the Table 2, the basic reference *EN*_*in*__*EN*_*out*_ achieves 0.80 ± 0.03 precision, 0.81 ± 0.05 recall, 0.80 ± 0.03 F1-score and 0.78 ± 0.05 accuracy for the classification. By introducing the decoders *DE*_*in*_ and *DE*_*out*_, the overall performance of the classification model is significantly improved. The optimal classification performance is generated from the whole NeAE_Eye model (*EN*_*in*__*DE*_*in*__*EN*_*out*__*DE*_*out*__*f*_*map*_). Compared with the basic reference model, the performance of the whole model is improved by 8.75, 9.87, 10.00, and 8.97% in mean precision, recall, F1-score and accuracy, respectively.

**Table 2:** Ablation experiment for the proposed NeAE-Eye model.

**Models**	**Mean precision**	**Mean recall**	**Mean F1-score**	**Mean accuracy**
*EN*_*in*__*EN*_*out*_	0.80 ± 0.03	0.81 ± 0.05	0.80 ± 0.03	0.78 ± 0.05
*EN*_*in*__*EN*_*out*__*DE*_*in*_	0.83 ± 0.05	0.85 ± 0.03	0.84 ± 0.05	0.80 ± 0.03
*EN*_*in*__*EN*_*out*__*DE*_*out*_	0.85 ± 0.06	0.88 ± 0.04	0.86 ± 0.05	0.81 ± 0.04
NeAE-Eye	0.87 ± 0.03	0.89 ± 0.04	0.88 ± 0.04	0.85 ± 0.05

**Figure 8:**
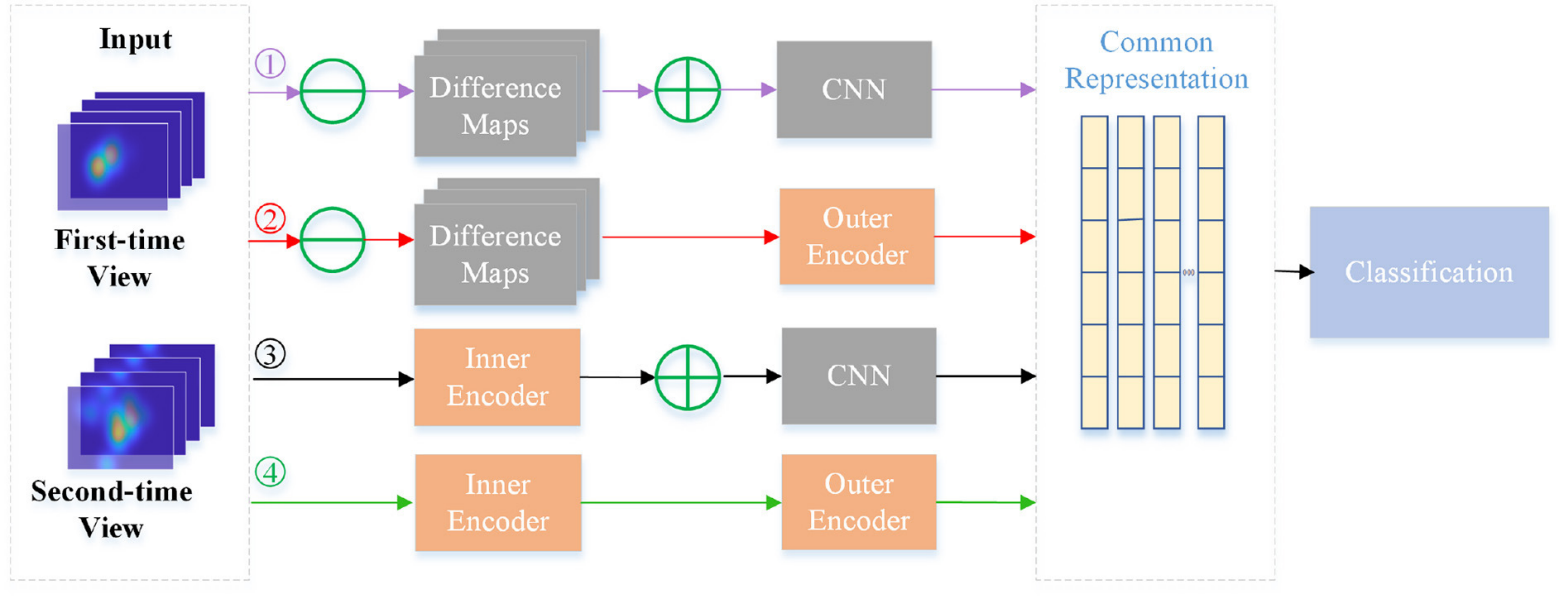
Flowchart of four different models for comparison. The four branches are: Model1 (Difference maps with CNN), Model2 (Difference maps with outer autoencoder), Model3 (Inner autoencoder with CNN) and Model4 (Inner autoencoder with outer autoencoder).

To further verify the effectiveness of inner and outer autoencoders in the NeAE-Eye model, we also design another ablation experiment by replacing them with other modules. For the inner autoencoder, we replace it with the difference maps, which are directly obtained by the difference of the corresponding fixation heatmaps for two-time views. As for the outer autoencoder, we change it to a shallow CNN network to extract subject-level common representation from the sum of total difference maps. As shown in Figure 8, there is a total of four models for comparison: Model1 (Difference maps with CNN), Model2 (Difference maps with outer autoencoder), Model3 (Inner autoencoder with CNN), and Model4 (Inner autoencoder with outer autoencoder). Moreover, the first nine layers of the VGG16 network is introduced as shallow CNN module.

In Table 3, we make a comparison of the experimental results of these four models. As this table shows, four models show different performances for classifying PwAD and HCs. Among them, the Model1 shows the worst performance, achieving 0.72± 0.02 precision, 0.74 ± 0.03 recall, 0.73 ± 0.03 F1-score and 0.71 ± 0.06 accuracy for the classification. By replacing the difference maps and CNN network with the inner autoencoder and outer autoencoder in turn, the classification accuracy of the two models (Model2 and Model3) is improved by 0.09 and 0.06, respectively. Model4, which combines the inner and outer autoencoders, shows the optimal performance for the classification.

**Table 3:** Ablation experiment with difference maps and CNN networks.

**Models**	**Mean precision**	**Mean recall**	**Mean F1-score**	**Mean accuracy**
Model1	0.72 ± 0.02	0.74 ± 0.03	0.73 ± 0.03	0.71 ± 0.06
Model2	0.82 ± 0.04	0.83 ± 0.03	0.82 ± 0.04	0.80 ± 0.04
Model3	0.80 ± 0.03	0.83 ± 0.05	0.81 ± 0.05	0.77 ± 0.05
Model4	0.87 ± 0.03	0.89 ± 0.04	0.88 ± 0.04	0.85 ± 0.05

The reasons for the differences in the performance of these models can be attributed to the following points: First, for the learning of image-dependent representation, the two branches of the inner autoencoder module can extract and fuse user's visual region preference features and novelty preference features, respectively. However, the difference map may confuse these two features by directly fusing the fixation heatmaps of the two-time views. Second, for the learning of subject-level representation, the adaptive weight fusion layer of the outer autoencoder can provide the classifier with more instructive features through continuous iterative learning. Assigning weights to each internal representation in an adaptive manner is more reasonable than summing them directly. Most importantly, autoencoders can retain more intrinsic information for feature learning in unsupervised processes compared with general CNN networks.

## 4. Discussion and the future work

The main purpose of this study is to investigate the potential clinical application of the machine-learning-based and deep-learning-based models on eye-tracking datasets in AD diagnosis. Judging from the fixation heatmaps generated by viewing the designed 3D VPC task, the PwAD group prefers to focus on the central area of the stereo scene and rarely on the RoI area where objects are added or removed. This result indicates that PwAD have a certain degree of memory decline and lack of novelty preference. These differential characteristics reflected in eye movement data between PwAD and HCs provide adequate support for clinical practice in diagnosing AD. As can be seen from Table 1, most models can make full use of these different features to classify AD and NC with good performance. Among them, the proposed NeAE-Eye model shows the best performance, of which the mean classification accuracy of a four-fold cross-validation experiment can reach 85%. Based on a nested autoencoder network, the proposed model can accurately extract the features of a single view, ensure the efficient fusion of features from different views and maximize the preservation of the original information of each view. Specifically, the inner autoencoder can comprehensively extract the eye movement features of a single view and the outer autoencoder is able to further deeply compresses the features from each view and guarantees that the final common representation provides more useful information for the final binary classification. In addition, the effect of each module in the overall model can be reflected in Table 2.

Although these findings require further validation in broader multi-source datasets, our analysis is sufficient to demonstrate that eye movement features under VPC tasks can serve as biomarkers for the clinical diagnosis of AD. Furthermore, applying deep-learning-based models can significantly improve diagnostic performance, as reporting can be improved by up to 17.99% in our study set. Future work will further improve the model's performance by considering more eye movement features, such as saccade and visual sensitivity. Besides, we will also optimize the network structure to achieve a refined classification of different degrees of cognitive impairment in AD. Moreover, we will also contribute to constructing a more comprehensive eye-movement dataset and exploring the different effects of 2D and 3D stimuli on classification.

## 5. Conclusions

In conclusion, this study validates the clinical utility of the proposed approach combined with eye-tracking technology to detect cognitive impairment in AD. The constructed large-scale eye-tracking dataset strongly reflects the differences in the eye-tracking characteristics of PwAD and HCs. Based on these differences, both the machine-learning-based and deep-learning-based approaches can achieve intelligent diagnosis of AD. Moreover, by introducing autoencoder networks with weight-adaptive fusion layers for feature extraction and feature fusion, the performance of the proposed NeAE-Eye model can be significantly improved.

## Data availability statement

The raw data supporting the conclusions of this article will be made available by the authors, without undue reservation.

## Ethics statement

The studies involving human participants were reviewed and approved by the Medical Ethics Committee of Tianjin Huanhu Hospital (ID:2020-60, approval 25 August 2020). The patients/participants provided their written informed consent to participate in this study.

## Author contributions

JS, YL, and HW contributed to the conception and design of the study. HW and YJ organized the database. JS, YL, and PJ performed the statistical analysis. JS and PJ wrote the first draft of the manuscript. HW wrote sections of the manuscript. All authors contributed to manuscript revision, read, and approved the submitted version.

## Funding

This research was funded by Yunnan provincial major science and technology special plan projects [202002AD080001]; the National Natural Science Foundation of China (61771338); Science and Technology Project of Tianjin Municipal Health Committee (ZC20121 and KJ20048).

## Conflict of interest

The authors declare that the research was conducted in the absence of any commercial or financial relationships that could be construed as a potential conflict of interest.

## Publisher's note

All claims expressed in this article are solely those of the authors and do not necessarily represent those of their affiliated organizations, or those of the publisher, the editors and the reviewers. Any product that may be evaluated in this article, or claim that may be made by its manufacturer, is not guaranteed or endorsed by the publisher.
